# Relationship between Body Composition and Pulmonary Function in Early Adult Life: A Cross-Sectional Analysis Nested in Two Birth Cohort Studies

**DOI:** 10.1371/journal.pone.0163428

**Published:** 2016-09-28

**Authors:** Paula Duarte de Oliveira, Fernando C. Wehrmeister, Rogelio Pérez-Padilla, Helen Gonçalves, Maria Cecília F. Assunção, Bernardo Lessa Horta, Denise P. Gigante, Fernando C. Barros, Ana Maria Baptista Menezes

**Affiliations:** 1 Federal University of Pelotas—Postgraduate Program in Epidemiology, Rua Marechal Deodoro, 1160, 3° andar, 96020–220, Pelotas, RS, Brazil; 2 National Institute of Respiratory Diseases, Tlalpan, 4502, 14080, Mexico City, DF, Mexico; 3 Catholic University of Pelotas—Postgraduate Program in Health and Behavior, Rua Gonçalves Chaves, 373 –sala 411, prédio C, 96015–560, Pelotas, RS, Brazil; Hunter College, UNITED STATES

## Abstract

**Background:**

Overweight/obesity has been reported to worsen pulmonary function (PF). This study aimed to examine the association between PF and several body composition (BC) measures in two population-based cohorts.

**Methods:**

We performed a cross-sectional analysis of individuals aged 18 and 30 years from two Pelotas Birth Cohorts in southern Brazil. PF was assessed by spirometry. Body measures that were collected included body mass index, waist circumference, skinfold thickness, percentages of total and segmented (trunk, arms and legs) fat mass (FM) and total fat-free mass (FFM). FM and FFM were measured by air-displacement plethysmography (BODPOD) and by dual-energy x-ray absorptiometry (DXA). Associations were verified through linear regressions stratified by sex, and adjusted for weight, height, skin color, and socioeconomic, behavioral, and perinatal variables.

**Results:**

A total of 7347 individuals were included in the analyses (3438 and 3909 at 30 and 18 years, respectively). Most BC measures showed a significant positive association between PF and FFM, and a negative association with FM. For each additional percentage point of FM, measured by BOD POD, the forced vital capacity regression coefficient adjusted by height, weight and skin color, at 18 years, was −33 mL (95% CI −38, −29) and −26 mL (95% CI −30, −22), and −30 mL (95% CI −35, −25) and −19 mL (95% CI −23, −14) at 30 years, in men and women, respectively. All the BOD POD regression coefficients for FFM were the same as for the FM coefficients, but in a positive trend (p<0.001 for all associations).

**Conclusions:**

All measures that distinguish FM from FFM (skinfold thickness–FM estimation–BOD POD, total and segmental DXA measures–FM and FFM proportions) showed negative trends in the association of FM with PF for both ages and sexes. On the other hand, FFM showed a positive association with PF.

## Introduction

Overweight/obesity is a growing risk factor in most countries worldwide [1]. This condition has been reported as an aggravating factor for many respiratory diseases or symptoms, such as obstructive sleep apnea, asthma, and dyspnea on effort, among others [2, 3]. Also, many studies have shown that obesity is associated to lower pulmonary function (PF) measures, even in non-diseased individuals [4, 5].

These findings have been attributed to the restriction and load imposed by excess fat mass (FM) on ventilatory mechanics [6, 7]. Fatty tissue may increase the thoracic cage load, and especially in supine decubitus, this places the diaphragm into a more expired and inefficient position [7]. Moreover, systemic inflammation owing to excess fat may cause airway inflammation and a consequent change in PF [6].

Even though there is an increasing number of studies on pulmonary function and body composition, mainly over the last decade, there are still unanswered questions, such as the role of fat-free mass (lean mass plus bone mineral content—FFM), which appears to be beneficial to PF due to its correlation with respiratory muscle strength [5, 8]. Additionally, identifying which body composition (BC) measures are more important for investigating the association of BC with PF would be useful.

Anthropometric measures, such as weight, waist circumference (WC), and body mass index (BMI), despite being widely used and provide some insight on BC and fat distribution (WC), are incapable to distinguish between FM and FFM and cannot precisely describe BC [4, 9]. These measures often fail to express the relation between BC and PF, particularly when confronted with measures which are able to differentiate body components [10, 11]. Few studies have assessed BC using higher precision methods, such as dual-energy x-ray absorptiometry (DXA) and air-displacement plethysmography (BOD POD). In addition, most of these studies targeted specific populations [6, 8, 10, 12–14], were carried out on volunteer samples [12, 13, 15], or evaluated healthcare patients [8, 10, 16], which limits data extrapolation to the general population.

The present study aimed to determine, in a population-based sample, the association between PF and several body measures, from the simplest and accessible anthropometric measures (such as BMI, WC and skinfolds) to the most complex ones obtained from more precise devices, such as BOD POD and DXA. We used data from 18- and 30-year-old individuals belonging to two birth cohorts.

## Material and Methods

This is a cross-sectional analysis carried out with data from two birth cohorts. In 1982 and 1993, mothers of all neonates born in Pelotas, a medium-sized city in southern Brazil, were invited to participate in the studies during the postpartum hospitalization. Mothers answered a perinatal questionnaire and agreed with undertaking some neonate tests. The last follow-up visits occurred in 2011 and 2012, when these individuals were 18 and 30 years old, respectively. The participants were invited to attend a clinic for the application of questionnaires and tests. More details on the methodology of these two cohort studies are described in previous publications [17, 18].

The present analysis included all participants who had spirometry performed. The exclusion criteria for the test, according to the participant’s report, were as follows: active tuberculosis, pregnancy, heart problems, thoracic, abdominal, or ocular surgery, and retinal displacement in the 3 previous months.

The spirometric variables that were analyzed were forced expiratory volume in the first second (FEV_1_) and forced vital capacity (FVC) prior to bronchodilator use. Both of these variables were assessed with a portable ultrasonic spirometer (EasyOne, Ndd Medical Technologies Inc., Zürich, Switzerland) and collected at 18 and 30 years as absolute values (liters). We followed the procedures recommended by the American Thoracic Society/European Respiratory Society [19], aiming for three acceptable maneuvers with maximum variation of 150 mL between the two best FVC and FEV_1_ values.

The exposure variables, collected in the same follow-ups, were anthropometric measures BMI (kg/m^2^), WC (measured in cm by trained personal using a tape measure in the narrowest point of the abdomen) and triceps and subscapular skinfold thickness (measured in mm using a plicometer, Cescorf Equipments, Porto Alegre, Brazil) and BC measures using the BOD POD (BOD POD® Composition System; COSMED, Albano Laziale, Italy) and DXA (model Lunar Prodigy Advance®; GE Healthcare, Freiburg, Germany), which yielded the percentages of FM and FFM. Furthermore, DXA was used to determine FM in the trunk, legs, and arms. The overall body FM and FFM percentages obtained through DXA were adjusted to the individuals’ body weight (FM or FFM/total mass×100).

The variables were described using mean and standard deviation for continuous variables and absolute and relative frequencies for categorical variables. The distribution of mean spirometric measurements in liters is shown using linear plots, stratified by sex and age, according to quintiles of BMI, WC, FM, and FFM (the last two variables were measured by the BOD POD). The associations between skinfold thickness and BC with FEV_1_ and FVC were determined using multivariate linear regression models stratified by sex and age, and were primarily adjusted by variables that were already established in the literature as predictors of PF[20]: height (cm, stadiometer—Harpenden, Holtain, Crymych, UK), weight (kg, BOD POD scale), and skin color (Model 1). Additional variables related to BC and PF, available in the cohort dataset, were selected as potential confounders and added to the Model 2. These variables were defined *a priori*: weight at birth and maternal smoking at any moment during pregnancy, collected during the perinatal interview; full years of schooling; family socioeconomic level (Asset Index in the 1993 Cohort and National Economic Index in the 1982 Cohort); smoking (never smoked, smoker, former smoker); self-reported wheezing over the previous year; corticosteroid use in the previous 3 months; and minutes of leisure physical activity, collected in the 2011 and 2012 follow-ups. The variation inflation factor (VIF) was verified to ensure the absence of collinearity among variables. No variable showed high multicollinearity (VIF < 10). The distribution of FEV_1_ and FVC linear regression coefficients in both models was also verified through quintiles for all anthropometric and body composition variables (available as additional tables). The analyses were performed using software Stata version 12.2 (Stata Corp., College Station, TX, USA). Values of p<0.05 in the Wald test for linear regression or heterogeneity were considered statistically significant.

All cohort follow-up projects were approved by the Federal University of Pelotas Ethics Committee. The follow-ups that were used in the present study were approved under protocols 05/11 and 16/12 for the 1993 and 1982 birth cohorts, respectively. The cohort participants, or their caregivers, signed the term of free and informed consent prior to participation.

## Results

The initial sample comprised 5914 and 5249 individuals born in 1982 and 1993, respectively. Several follow-ups were carried out at different ages. The overall cohort follow-up rates in 2011 and 2012 were 68.1% (3701 individuals who attended to the clinic plus 325 deaths) and 81.3% (4106 individuals who attended to the clinic plus 164 deaths) for those born in 1982 and 1993, respectively. These follow-up rates are attributed to loss of contact or refusal to continue to participate in the cohort studies. To our specific study, a total of 7347 individuals who underwent spirometry were included in the analyses (3438 and 3909 from the 1982 and 1993 cohorts, respectively); 88.5% and 91.6% of the tests at 18 and 30 years, respectively, met the quality criteria according to the American Thoracic Society guidelines [19].

The characteristics of the individuals who were included in the analyses were similar to those of the total of cohort participants regarding sex and socioeconomic level in both cohorts. This ensured the representativeness of the general population (data not shown in tables).

A description of the sample, according to the current and perinatal variables, as well as the anthropometric characteristics, BC, and PF at 18 and 30 years, are shown in Table 1. Most participants were white and had 9 or more years of schooling, except for male adolescents. The mean of most of the anthropometric variables (except for triceps skinfold) and percentage of FM were higher in individuals of the 1982 cohort than in those of the 1993 cohort. The opposite finding was observed for the percentage of FFM. The mean FEV_1_ and FVC values for both cohorts were similar, with approximately 4.0 L FEV_1_ in men and 3.0 L FEV_1_ in women. Mean FVC was 4.8 L in men and 3.5 L in women (Table 1).

**Table 1:** Description of the sample as covariates, body composition and lung function.

	1993 Cohort—18 years		1982 Cohort—30 years	
	(n = 3,909)		(n = 3,438)	
Social, demographic and behavioral variables	Male	Female	Male	Female
	(n = 1,964)	(n = 1,945)	(n = 1,717)	(n = 1,721)
	N (%)	N (%)	N (%)	N (%)
**Birth weight (grams)**^**1**^				
**> = 2500g**	1,815 (92.4)	1,741 (89.5)	1,614 (94.0)	1,572 (91.4)
**<2500g**	149 (7.6)	204 (10.5)	103 (6.0)	148 (8.6)
**Maternal smoking during pregnancy**^**1**^				
**No**	1,332 (67.8)	1,292 (66.4)	1,115 (64.9)	1,120 (65.1)
**Yes**	632 (32.2)	653 (33.6)	602 (35.1)	601 (34.9)
**Skin color**				
**White**	1,274 (67.8)	1,248 (65.8)	1,290 (75.1)	1,315 (76.4)
**Black**	239 (12.7)	240 (12.7)	274 (16.0)	274 (15.9)
**Brown**	278 (14.8)	321 (16.9)	95 (5.5)	80 (4.7)
**Others**	87 (4.7)	88 (4.7)	58 (3.4)	52 (3.0)
**Socioeconomic status (quintiles)**^**2**^				
**1 (poorest)**	329 (16.8)	452 (23.3)	338 (21.2)	403 (24.9)
**2**	395 (20.2)	383 (19.7)	342 (21.4)	339 (20.9)
**3**	391 (20.0)	389 (20.0)	442 (27.7)	414 (25.5)
**4**	423 (21.6)	357 (18.4)	152 (9.5)	155 (9.6)
**5 (richest)**	418 (21.4)	360 (18.6)	324 (20.3)	310 (19.1)
**Education (years)**				
**0–4**	130 (6.7)	53 (2.7)	103 (6.1)	102 (6.0)
**5–8**	922 (47.1)	645 (33.2)	381 (22.4)	290 (17.0)
**9–11**	847 (43.3)	1,151 (59.3)	551 (32.5)	481 (28.2)
**≥ 12**	57 (2.9)	92 (4.7)	662 (39.0)	833 (48.8)
**Smoking status**				
**Never**	1,511 (77.2)	1,524 (78.5)	978 (57.0)	1,040 (60.5)
**Former**	145 (7.4)	163 (8.4)	296 (17.2)	312 (18.2)
**Smoker**	302 (15.4)	254 (13.1)	443 (25.8)	366 (21.3)
**Wheezing in the last year**				
**No**	1,735 (88.6)	1,666 (85.8)	1,488 (86.7)	1,437 (83.5)
**Yes**	223 (11.4)	275 (14.2)	229 (13.3)	284 (16.5)
**Corticoids use in the last three months**^**3**^				
**No**	1,933 (98.4)	1,900 (97.5)	1,576 (95.5)	1,480 (89.9)
**Yes**	31 (1.6)	45 (2.5)	75 (4.5)	167 (10.1)
**Leisure physical activity** ^**4**^				
**Inactive**	1058 (54.2)	1,507 (77.8)	1,040 (61.7)	1,341 (79.0)
**Active**	895 (45.8)	431 (22.2)	646 (38.3)	356 (21.0)
**Anthropometric, body composition and lung function variables**	Mean (SD)	Mean (SD)	Mean (SD)	Mean (SD)
**Height (cm)**	173.8 (6.9)	161.1 (6.5)	174.4 (6.9)	161.4 (6.2)
**Weight (kg)**	70.7 (14.3)	61.1 (13.1)	82.2 (16.8)	69.6 (16.2)
**BMI (kg/m²)**	23.4 (4.2)	23.5 (4.8)	27.0 (5.0)	26.7 (6.0)
**Waist circumference (cm)**	78.4 (9.6)	73.6 (9.7)	89.2 (11.7)	80.6 (12.0)
**Triceps skinfold (mm)**	12.1 (7.6)	21.9 (9.0)	10.1 (5.2)	17.8 (8.5)
**Subscapular skinfold (mm)**	11.9 (5.6)	16.1 (7.6)	18.2 (7.5)	21.3 (8.7)
**Fat mass BOD POD (%)**	16.8 (8.9)	32.6 (7.9)	24.5 (9.2)	37.4 (8.5)
**Fat- free mass BOD POD (%)**	83.2 (8.9)	67.4 (7.9)	75.5 (9.2)	62.7 (8.5)
**Fat mass DXA (% adjusted)**	16.9 (9.4)	34.8 (8.5)	24.2 (8.7)	39.2 (8.5)
**Fat-free mass DXA (% adjusted)**	78.2 (8.7)	61.7 (8.1)	71.4 (8.0)	57.7 (8.0)
**Trunk fat mass DXA (%)**	18.9 (10.7)	35.9 (9.4)	28.9 (10.3)	40.7 (9.4)
**Arms fat mass DXA (%)**	11.2 (7.8)	29.0 (8.6)	16.5 (7.7)	33.2 (8.8)
**Legs fat mass DXA (%)**	17.6 (9.3)	37.7 (8.1)	22.0 (7.9)	41.4 (8.0)
**FEV**_**1**_ **(L)**	4.1 (0.6)	3.0 (0.5)	4.0 (0.6)	2.9 (0.5)
**FVC (L)**	4.8 (0.7)	3.5 (0.5)	4.8 (0.8)	3.5 (0.5)

N: number of observations; SD: standard deviation; BMI: body mass index; DXA: dual-energy X-ray absorptiometry; BOD POD: air displacement plethysmography; FEV_1_: forced expiratory volume in the first second; FVC: forced vital capacity

^1^Variables collected in the perinatal follow up, other variables collected at 18 and 30.

^2^Socioeconomic status by the Asset Index at age 18 (1993 Cohort) and National Economic Index (IEN) at 30 (1982 Cohort)

^3^Maximum number of missing values: 136 observations in corticoids use.

^4^Cutoff points for physical activity 300 minutes/week at 18 and 150 minutes/week at age 30, as recommended by the World Health Organization for adolescents and adults.

**Fig 1:**
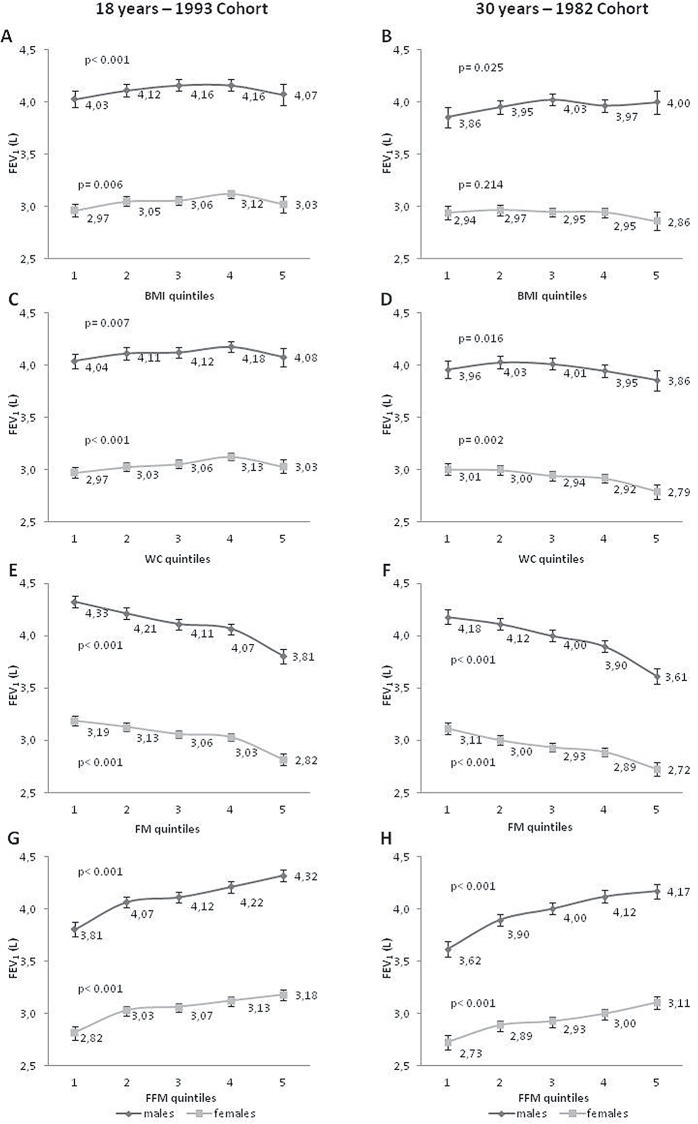
FEV_1_ means (95%CI) distribution according to quintiles of BMI, WC, fat mass and fat free mass. FEV_1_: forced expiratory volume in the first second; BMI: body mass index; WC: waist circumference; FM: fat mass; FFM fat free mass. Adjusted by height, weight and skin color current asset index (quintiles), current achieved schooling, smoking status, wheezing in the last year, physical activity and corticoids use in the last three months, birth weight and maternal smoking during pregnancy. P-values for A–D plots: Wald’s test for heterogeneity; p-values for E—H plots: Wald’s test for linear tendency. First quintile: lowest values; fifth quintile: highest values (anthropometric and body composition variables)

**Fig 2:**
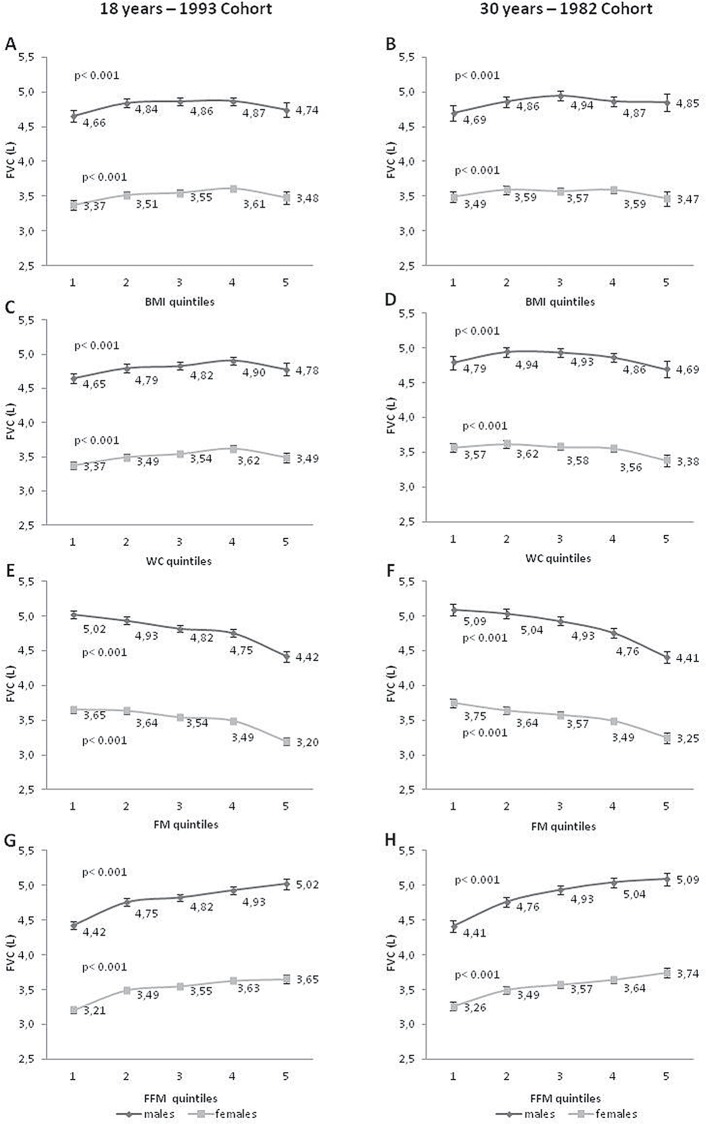
FVC means (95%CI) distribution according to quintiles of BMI, WC, fat mass and fat free mass. FVC: forced vital capacity; BMI: body mass index; WC: waist circumference; FM: fat mass; FFM fat free mass. p-values for A–D plots: Wald’s test for heterogeneity; ADJUSTED by height, weight and skin color current asset index (quintiles), current achieved schooling, smoking status, wheezing in the last year, physical activity and corticoids use in the last three months, birth weight and maternal smoking during pregnancy. P-values for E—H plots: Wald’s test for linear tendency. First quintile: lowest values; fifth quintile: highest values (anthropometric and body composition variables)

Figs 1 and 2 show the distribution of the mean FEV_1_ and FVC (L) according to BMI, WC, FM, and FFM in quintiles. At 18 years, we observed a slight upward trend in both spirometric parameters from the 1^st^ to the 4^th^ quintile, and then a slight decrease trend from the 4^th^ to the 5^th^ quintile, for BMI and WC. At the age of 30 years, there was no clear pattern regarding these two measures. However, there was a consistent pattern for the associations of FM and FFM and the two parameters of PF in both cohorts in male and female individuals. An inverse linear relationship between FM and PF and a positive linear association for FFM and PF parameters were observed.

Tables 2–5 show multivariable linear regression analysis for the associations among PF parameters and skinfold thickness and BC measures, and male (Tables 2 and 4) and female (Tables 3 and 5) individuals, in both cohorts. For FEV_1_, most of the linear associations that were assessed were significant; FEV_1_ was negatively associated with FM, independent of the measurement methods of BC. Conversely, FEV_1_ and the FFM percentage were positively associated at both ages. There was no association between FEV_1_ and DXA for 30-year-old women in Model 1 (Table 3). However, when we performed an analysis adjusted for several other confounders (Model 2: weight at birth, maternal smoking during pregnancy, years of schooling, family socioeconomic level, smoking, self-reported wheezing, corticosteroid use, and minutes of leisure physical activity), all of the variables reached significance, showing the same trends, except for the percentage of trunk fat (Table 3).

**Table 2:** Association between FEV_1_ (L) and anthropometric variables and body composition, males, at 18 and 30 years.

	1993 Cohort—18 years		1982 Cohort—30 years	
	Model 1*	Model 2**	Model 1*	Model 2**
	β (95% CI)	β (95% CI)	β (95% CI)	β (95% CI)
**Triceps skinfold (mm)**	p <0.001	p <0.001	p = 0.025	p <0.001
	-0.020 (-0.025; -0.015)	-0.020 (-0.025; -0.015)	-0.006 (-0.011; -0.001)	-0.007 (-0.012; -0.001)
**Subscapular skinfold (mm)**	p <0.001	p <0.001	p <0.001	p <0.001
	-0.023 (-0.030; -0.016)	-0.022 (-0.030; -0.015)	-0.022 (-0.028; -0.017)	-0.022 (-0.028; -0.017)
**Fat mass BOD POD (%)**	p <0.001	p <0.001	p <0.001	p <0.001
	-0.027 (-0.031; -0.022)	-0.027 (-0.031; -0.022)	-0.023 (-0.028; -0.019)	-0.024 (-0.028; -0.019)
**Fat-free mass BOD POD (%)**	p <0.001	p <0.001	p <0.001	p <0.001
	0.027 (0.022; 0.031)	0.027 (0.022; 0.031)	0.023 (0.019; 0.028)	0.024 (0.019; 0.028)
**Fat mass DXA (% adjusted)**	p <0.001	p <0.001	p <0.001	p <0.001
	-0.015 (-0.019; -0.010)	-0.016 (-0.021; -0.011)	-0.016 (-0.021; -0.011)	-0.018 (-0.023; -0.013)
**Fat- free mass DXA (% adjusted)**	p <0.001	p <0.001	p <0.001	p <0.001
	0.016 (0.011; 0.021)	0.017 (0.012; 0.022)	0.017 (0.012; 0.022)	0.019 (0.014; 0.024)
**Trunk fat mass DXA (%)**	p <0.001	p <0.001	p <0.001	p <0.001
	-0.012 (-0.016; -0.008)	-0.014 (-0.018; -0.010)	-0.013 (-0.017; -0.009)	-0.015 (-0.019; -0.011)
**Arms fat mass DXA (%)**	p <0.001	p <0.001	p <0.001	p <0.001
	-0.018 (-0.023; -0.013)	-0.018 (-0.024; -0.013)	-0.019 (-0.024; -0.013)	-0.021 (-0.026; -0.015)
**Legs fat mass DXA (%)**	p <0.001	p <0.001	p <0.001	p <0.001
	-0.012 (-0.016; -0.008)	-0.013 (-0.017; -0.009)	-0.013 (-0.018; -0.009)	-0.014 (-0.019; -0.010)

β: regression coefficient; CI: confidence interval; BMI: body mass index; WC: waist circumference; DXA: dual-energy X-ray absorptiometry; BOD POD: air displacement plethysmography; FEV_1_: forced expiratory volume in the first second; DXA variables 18 years: 1778 observations. DXA variables 30 years: 1638 observations.

* Model 1: adjusted by height, weight and skin color (18 years n = 1844 / 30 years n = 1711)

**Model 2: adjusted by Model 1 + current asset index (quintiles), current achieved schooling, smoking status, wheezing in the last year, physical activity and corticoids use in the last three months, birth weight and maternal smoking during pregnancy (18 years n = 1833/30 years n = 1593).

P-value: Wald’s test for linear tendency.

**Table 3:** Association between FEV_1_ (L) and anthropometric variables and body composition, females, at 18 and 30 years.

	1993 Cohort—18 years		1982 Cohort—30 years	
	Model 1*	Model 2**	Model 1*	Model 2**
	β (95% CI)	β (95% CI)	β (95% CI)	β (95% CI)
**Triceps skinfold (mm)**	p <0.001	p <0.001	p = 0.024	p = 0.001
	-0.010 (-0.014; -0.007)	-0.012 (-0.016; -0.009)	-0.003 (-0.006; -0.0004)	-0.004 (-0.007; -0.002)
**Subscapular skinfold (mm)**	p <0.001	p <0.001	p <0.001	p <0.001
	-0.010 (-0.014; -0.007)	-0.010 (-0.014; -0.006)	-0.009 (-0.013; -0.006)	-0.008 (-0.012; -0.005)
**Fat mass BOD POD (%)**	p <0.001	p <0.001	p <0.001	p <0.001
	-0.020 (-0.023; -0.016)	-0.021 (-0.025; -0.018)	-0.013 (-0.017; -0.009)	-0.016 (-0.020; -0.012)
**Fat-free mass BOD POD (%)**	p <0.001	p <0.001	p <0.001	p <0.001
	0.020 (0.016; 0.023)	0.021 (0.018; 0.025)	0.013 (0.009; 0.017)	0.016 (0.012; 0.020)
**Fat mass DXA (% adjusted)**	p <0.001	p <0.001	p = 0.407	p = 0.018
	-0.011 (-0.014; -0.007)	-0.013 (-0.017; -0.010)	-0.002 (-0.005; 0.002)	-0.004 (-0.008; -0.001)
**Fat-free mass DXA (% adjusted)**	p <0.001	p <0.001	p = 0.558	p = 0.037
	0.011 (0.007; 0.015)	0.014 (0.010; 0.017)	0.001 (-0.003; 0.005)	0.004 (0.0002; 0.008)
**Trunk fat mass DXA (%)**	p <0.001	p <0.001	p = 0.301	p = 0.084
	-0.009 (-0.012; -0.006)	-0.011 (-0.014; -0.007)	-0.002 (-0.005; 0.002)	-0.003 (-0.006; 0.0004)
**Arms fat mass DXA (%)**	p <0.001	p <0.001	p = 0.103	p <0.001
	-0.007 (-0.010; -0.004)	-0.010 (-0.014; -0.007)	-0.003 (-0.006; 0.001)	-0.007 (-0.011; -0.003)
**Legs fat mass DXA (%)**	p <0.001	p <0.001	p = 0.869	p = 0.048
	-0.009 (-0.012; -0.006)	-0.012 (-0.015; -0.008)	0.0003 (-0.003; 0.004)	-0.003 (-0.007; -0.00003)

β: regression coefficient; CI: confidence interval; BMI: body mass index; WC: waist circumference; DXA: dual-energy X-ray absorptiometry; BOD POD: air displacement plethysmography; FEV_1_: forced expiratory volume in the first second; DXA variables 18 years: 1888 observations. DXA variables 30 years: 1700 observations.

* Model 1: adjusted by height, weight and skin color (18 years n = 1900 / 30 years n = 1715)

**Model 2: adjusted by Model 1 + current asset index (quintiles), current achieved schooling, smoking status, wheezing in the last year, physical activity and corticoids use in the last three months, birth weight and maternal smoking during pregnancy (18 years n = 1896 / 30 years n = 1612).

P-value: Wald’s test for linear tendency.

**Table 4:** Association between FVC (L) and anthropometric variables and body composition, males, at 18 and 30 years.

	1993 Cohort—18 years		1982 Cohort—30 years	
	Model 1*	Model 2**	Model 1*	Model 2**
	β (95% CI)	β (95% CI)	β (95% CI)	β (95% CI)
**Triceps skinfold (mm)**	p <0.001	p <0.001	p = 0.006	p = 0.003
	-0.028 (-0.034; -0.023)	-0.027 (-0.033; -0.022)	-0.008 (-0.014; -0.002)	-0.009 (-0.015; -0.003)
**Subscapular skinfold (mm)**	p <0.001	p <0.001	p <0.001	p <0.001
	-0.034 (-0.042; -0.026)	-0.032 (-0.040; -0.024)	-0.031 (-0.037; -0.024)	-0.029 (-0.036; -0.023)
**Fat mass BOD POD (%)**	p <0.001	p <0.001	p <0.001	p <0.001
	-0.033 (-0.038; -0.029)	-0.033 (-0.038; -0.029)	-0.030 (-0.035; -0.025)	-0.029 (-0.034; -0.024)
**Fat-free mass BOD POD (%)**	p <0.001	p <0.001	p <0.001	p <0.001
	0.033 (0.029; 0.038)	0.033 (0.029; 0.038)	0.030 (0.025; 0.035)	0.029 (0.024; 0.035)
**Fat mass DXA (% adjusted)**	p <0.001	p <0.001	p <0.001	p <0.001
	-0.021 (-0.026; -0.017)	-0.022 (-0.027; -0.017)	-0.020 (-0.026; -0.015)	-0.020 (-0.026; -0.015)
**Fat-free mass DXA (% adjusted)**	p <0.001	p <0.001	p <0.001	p <0.001
	0.023 (0.018; 0.028)	0.024 (0.019; 0.030)	0.021 (0.016; 0.027)	0.022 (0.016; 0.028)
**Trunk fat mass DXA (%)**	p <0.001	p <0.001	p <0.001	p <0.001
	-0.018 (-0.022; -0.014)	-0.019 (-0.024; -0.015)	-0.017 (-0.022; -0.012)	-0.017 (-0.022; -0.013)
**Arms fat mass DXA (%)**	p <0.001	p <0.001	p <0.001	p <0.001
	-0.025 (-0.031; -0.020)	-0.025 (-0.031; -0.020)	-0.024 (-0.030; -0.018)	-0.025 (-0.031; -0.019)
**Legs fat mass DXA (%)**	p <0.001	p <0.001	p <0.001	p <0.001
	-0.017 (-0.022; -0.013)	-0.018 (-0.022; -0.014)	-0.016 (-0.021; -0.010)	-0.015 (-0.021; -0.010)

β: regression coefficient; CI: confidence interval; BMI: body mass index; WC: waist circumference; DXA: dual-energy X-ray absorptiometry; BOD POD: air displacement plethysmography; FVC: forced vital capacity; DXA variables 18 years: 1778 observations. DXA variables 30 years: 1638 observations.

* Model 1: adjusted by height, weight and skin color (18 years n = 1844 / 30 years n = 1711)

**Model 2: adjusted by Model 1 + current asset index (quintiles), current achieved schooling, smoking status, wheezing in the last year, physical activity and corticoids use in the last three months, birth weight and maternal smoking during pregnancy (18 years n = 1833/30 years n = 1593).

P-value: Wald’s test for linear tendency.

**Table 5:** Association between FVC (L) and anthropometric variables and body composition, females, at 18 and 30 years.

	1993 Cohort—18 years		1982 Cohort—30 years	
	Model 1*	Model 2**	Model 1*	Model 2**
	β (95% CI)	β (95% CI)	β (95% CI)	β (95% CI)
**Triceps skinfold (mm)**	p < 0.001	p < 0.001	0.006	p <0.001
	-0.015 (-0.019; -0.012)	-0.017 (-0.020; -0.013)	-0.004 (-0.007; -0.001)	-0.005 (-0.008; -0.002)
**Subscapular skinfold (mm)**	p < 0.001	p < 0.001	p < 0.001	p <0.001
	-0.016 (-0.020; -0.012)	-0.015 (-0.019; -0.011)	-0.013 (-0.017; -0.009)	-0.012 (-0.016; -0.008)
**Fat mass BOD POD (%)**	p < 0.001	p < 0.001	p < 0.001	p <0.001
	-0.026 (-0.030; -0.022)	-0.027 (-0.031; -0.023)	-0.019 (-0.023; -0.014)	-0.021 (-0.026; -0.017)
**Fat-free mass BOD POD (%)**	p < 0.001	p < 0.001	p < 0.001	p <0.001
	0.026 (0.022; 0.030)	0.027 (0.023; 0.031)	0.019 (0.014; 0.023)	0.021 (0.017; 0.026)
**Fat mass DXA (% adjusted)**	p < 0.001	p < 0.001	p = 0.031	p = 0.003
	-0.016 (-0.020; -0.012)	-0.017 (-0.021; -0.013)	-0.004 (-0.009; -0.0004)	-0.007 (-0.011; -0.002)
**Fat-free mass DXA (% adjusted)**	p < 0.001	p < 0.001	p = 0.074	p = 0.009
	0.017 (0.013; 0.021)	0.018 (0.014; 0.022)	0.004 (-0.0004; 0.008)	0.006 (0.001; 0.010)
**Trunk fat mass DXA (%)**	p < 0.001	p < 0.001	p = 0.039	p = 0.022
	-0.014 (-0.017; -0.010)	-0.014 (-0.017; -0.011)	-0.004 (-0.007; -0.0002)	-0.004 (-0.008; -0.001)
**Arms fat mass DXA (%)**	p < 0.001	p < 0.001	p < 0.001	p <0.001
	-0.013 (-0.017; -0.010)	-0.015 (-0.019; -0.012)	-0.008 (-0.012; -0.004)	-0.012 (-0.016; -0.008)
**Legs fat mass DXA (%)**	p < 0.001	p < 0.001	p = 0.303	p = 0.021
	-0.013 (-0.016; -0.010)	-0.014 (-0.018; -0.011)	-0.002 (-0.006; 0.002)	-0.005 (-0.008; -0.001)

β: regression coefficient; CI: confidence interval; BMI: body mass index; WC: waist circumference; DXA: dual-energy X-ray absorptiometry; BOD POD: air displacement plethysmography; FVC: forced vital capacity. DXA variables 18 years: 1888 observations. DXA variables 30 years: 1700 observations.

* Model 1: adjusted by height, weight and skin color (18 years n = 1900 / 30 years n = 1715)

**Model 2: adjusted by Model 1 + current asset index (quintiles), current achieved schooling, smoking status, wheezing in the last year, physical activity and corticoids use in the last three months, birth weight and maternal smoking during pregnancy (18 years n = 1896 / 30 years n = 1612).

P-value: Wald’s test for linear tendency.

For FVC (Tables 4 and 5), similar results to those for FEV_1_ were observed. In 30-year-old women, the percentages of FFM measured by DXA and FM in the lower limbs were not associated with FVC in the Model 1. However, a significant beta was observed when model 2 was applied (Table 5).

The distribution of PF regression coefficients and all the anthropometric and BC variables by quintiles, adjusted by models 1 and 2, is available as supplementary material. According to the FM measured by BOD POD, someone aged 30 years belonging to the fifth quintile (highest) has a mean decrease of FVC 680ml and 480ml, for men and women, respectively, compared to those who are in the first quintile (S1–S4 Tables).

## Discussion

Our findings showed that FM was inversely associated with PF, particularly in men. In addition, this association was in the opposite direction for FFM levels.

BMI and WC, which are widely used in epidemiological studies, yielded inconsistent results for 18 and 30 years in our study, not showing the same trends for different ages (18 and 30 years) and sexes. This finding may be due to the fact that these two anthropometric measures do not distinguish fat from FFM. Usually a high BMI means high adiposity levels, but a person with high FFM can also have an elevated BMI. The same can happen with WC that is usually recommended as measure of central obesity, mainly in young adults [21]. Additionally, all results for FM and FFM showed opposite trends in the association with PF, which may explain the absence of association pattern in measures that cannot distinguish these two components. These two anthropometric measures (BMI and WC) were removed from the linear regression analysis because their relationship with PF was not linear and it could lead to incorrect interpretations. Previous studies have shown that the relation between BMI and PF is not linear at several ages [11, 22–25], and this was observed in our study (Figs 1 and 2). One example of such a non-linear relation is from a study carried out by Fogarty et al. [26], who showed a reduction in FEV_1_ by 122 mL (95% CI −234, −10) in those with a BMI below 20 kg/m^2^ and by 85 mL (95% CI −160, −9) in those with a BMI above 30 kg/m^2^ compared with individuals with a normal BMI.

In the current study, FM and FFM, measured by the BOD POD, had the highest linear regression coefficients among the BC measures and PF for both sexes compared with the measures obtained by DXA. They also had the highest adjusted r^2^ values in both adjusted models (data not shown).

Although the mean FEV_1_ and FVC were similar in individuals at 18 and 30 years, the regression coefficients of the association between BC and PF tended to be lower at 30 years, particularly in women, when compared to 18 years old individuals. This finding suggests that other factors, such as smoking and/or comorbidities, play a greater role in PF at 30 years. Our results showed a negative confounding after adjustment to variables such as wheezing, corticoid use and smoking status, strengthening most of the associations in relation to Model 1, mainly among women and at age 30 years. Findings in the literature are less consistent for women than for men, mainly regarding the associations between FFM and PF [5, 6, 12, 15, 27–30]. One possible explanation for this difference in the previous studies could be the lack of adjustment for important confounding factors in the assessment of adults, such as those used in Model 2 that changed the results between DXA and PF for 30-year-old women, turning most of the associations from non-significant into significant.

Notably, DXA tends to overestimate the FM percentage in women compared with the BOD POD [31], and this has been also observed in our study (described in Table 1). This could also explain the inconsistent results among previous studies with DXA [5, 6, 15]. Only one of these studies [5] used DXA in a population-based sample. Most PF measures, particularly FVC, total lung capacity, and residual functional capacity, were inversely correlated to FM measures from DXA and anthropometric measures, which are consistent with the present findings. With regard to FFM, Sutherland et al. found a positive association with PF only for male individuals, while in the present study, this association was found for both sexes. To the best of our knowledge, no studies used the BOD POD to determine the association between BC and PF.

Previous studies also found an association of the distribution of FM on PF [5, 6, 14, 25, 29, 32–39]. In the present study, WC was not linearly associated with PF at 18 and 30 years old. Other studies with adolescents also found no associations between WC and FEV_1_ or FVC, or found a positive association [37–39], while studies with adults found an inverse association of WC with PF [5, 6, 25, 28, 32–36]. A recent study found WC as a predictor of reduced FVC after adjustment for several potential confounders, in adult women after 10 years of follow-up [40]. In our study, women aged 30 years also tend to have decreased FEV_1_ and FVC, when comparing the fifth quintile of WC to the first, mainly after the adjustment for Model 2. Trunk fat was inversely associated with the PF parameters in studies that assessed BC by segments [5, 10–12, 14], an expected result because of the restriction in ventilatory mechanics [6, 7]. However, in the present analyses, FM in the arms, instead of trunk fat, was the segment most strongly associated with PF. This finding is in accordance with other studies that showed an inverse association of arm fat with PF [6, 41]. Scott et al. [6] showed that the correlation between FM in the arms and FVC was r = −0.753, and the correlation with FEV_1_ was r = −0.535. These results are attributed to the hypothesis that accumulation of FM in the upper body is more harmful to PF than that in the lower body [6, 41]. Our results for triceps and subscapular skinfolds are consistent with this hypothesis.

Some strengths of the present study are as follows. We used a representative sample with a large number of participants, which confers power to detect associations. We had a high follow-up rate of the cohort members and rigorous quality control in spirometry. We used highly precise techniques to assess BC. Finally, there was the possibility of adjustment for several confounding factors that were available since the perinatal follow-ups. This study also has some limitations. We did not have the information about pack-years of smoking to estimate smoke load that could be a more accurate variable for adjustment. We did not have any static lung volume measures, such as total lung capacity and expiratory reserve volume. In addition, the impossibility of performing longitudinal analyses of BC does not allow temporality to be established between body measures and PF. Although spirometric and anthropometric measurements were performed in previous follow-ups, measurements using DXA and the BOD POD were only carried out in the latest cohort follow-ups. Longitudinal analyses using data of future follow-ups will contribute to a better understanding of associations.

## Conclusions

We conclude that all body measures that distinguish FM from FFM, either through more accessible methods, such as skinfold thickness, giving FM estimation, or by sophisticated devices (DXA or BOD POD, giving both FM and FFM proportions), show the same trends in the results with consistent associations with PF. BMI and WC, that cannot distinguish body components do not show a pattern of association with PF; while greater FM levels show linear and negative associations with FEV_1_ and FVC. Higher levels of FFM seem to raise these parameters. These findings occur at 18 and 30 years of age for both sexes, thus strengthening our results.

## Supporting Information

S1 Table Association between FEV_1_ and anthropometric variables and body composition (quintiles), males, at 18 and 30 years. β: regression coefficient; CI: confidence interval; BMI: body mass index; WC: waist circumference; DXA: dual-energy X-ray absorptiometry; BOD POD: air displacement plethysmography; FEV_1_: forced expiratory volume in the first second; DXA variables 18 years: 1778 observations. DXA variables 30 years: 1638 observations. * Model 1: adjusted by height, weight and skin color (18 years n = 1844 / 30 years n = 1711). **Model 2: adjusted by Model 1 + current asset index (quintiles), current achieved schooling, smoking status, wheezing in the last year, physical activity and corticoids use in the last three months, birth weight and maternal smoking during pregnancy (18 years n = 1833/30 years n = 1593). P-value: Wald’s test for linear tendency, except BMI and WC Wald’s test for heterogeneity.
(DOCX)

S2 Table Association between FEV_1_ and anthropometric variables and body composition (quintiles), females, at 18 and 30 years. β: regression coefficient; CI: confidence interval; BMI: body mass index; WC: waist circumference; DXA: dual-energy X-ray absorptiometry; BOD POD: air displacement plethysmography; FEV_1_: forced expiratory volume in the first second; DXA variables 18 years: 1888 observations. DXA variables 30 years: 1700 observations. * Model 1: adjusted by height, weight and skin color (18 years n = 1900 / 30 years n = 1715). **Model 2: adjusted by Model 1 + current asset index (quintiles), current achieved schooling, smoking status, wheezing in the last year, physical activity and corticoids use in the last three months, birth weight and maternal smoking during pregnancy (18 years n = 1896 / 30 years n = 1612). P-value: Wald’s test for linear tendency, except BMI and WC Wald’s test for heterogeneity.
(DOCX)

S3 Table Association between FVC and anthropometric variables and body composition (quintiles), males, at 18 and 30 years. β: regression coefficient; CI: confidence interval; BMI: body mass index; WC: waist circumference; DXA: dual-energy X-ray absorptiometry; BOD POD: air displacement plethysmography; FVC: forced vital capacity; DXA variables 18 years: 1778 observations. DXA variables 30 years: 1638 observations. * Model 1: adjusted by height, weight and skin color (18 years n = 1844 / 30 years n = 1711). **Model 2: adjusted by Model 1 + current asset index (quintiles), current achieved schooling, smoking status, wheezing in the last year, physical activity and corticoids use in the last three months, birth weight and maternal smoking during pregnancy (18 years n = 1833/30 years n = 1593). P-value: Wald’s test for linear tendency, except BMI and WC Wald’s test for heterogeneity.
(DOCX)

S4 Table Association between FVC and anthropometric variables and body composition, females, at 18 and 30 years. β: regression coefficient; CI: confidence interval; BMI: body mass index; WC: waist circumference; DXA: dual-energy X-ray absorptiometry; BOD POD: air displacement plethysmography; FVC: forced vital capacity. DXA variables 18 years: 1888 observations. DXA variables 30 years: 1700 observations. * Model 1: adjusted by height, weight and skin color (18 years n = 1900 / 30 years n = 1715). **Model 2: adjusted by Model 1 + current asset index (quintiles), current achieved schooling, smoking status, wheezing in the last year, physical activity and corticoids use in the last three months, birth weight and maternal smoking during pregnancy (18 years n = 1896 / 30 years n = 1612). P-value: Wald’s test for linear tendency, except BMI and WC Wald’s test for heterogeneity.
(DOCX)
