# Supplementary material for: Relationship between Body Composition and Pulmonary Function in Early Adult Life: A Cross-Sectional Analysis Nested in Two Birth Cohort Studies
Source: PLoS One. 2016 Sep 28;11(9):e0163428. doi: 10.1371/journal.pone.0163428 (PMC5040394; doi:10.1371/journal.pone.0163428)
Supplement: S3 Table — β: regression coefficient; CI: confidence interval; BMI: body mass index; WC: waist circumference; DXA: dual-energy X-ray absorptiometry; BOD POD: air displacement plethysmography; FVC: forced vital capacity; DXA variables 18 years: 1778 observations. DXA variables 30 years: 1638 observations. * Model 1: adjusted by height, weight and skin color (18 years n = 1844 / 30 years n = 1711). **Model 2: adjusted by Model 1 + current asset index (quintiles), current achieved schooling, smoking status, wheezing in the last year, physical activity and corticoids use in the last three months, birth weight and maternal smoking during pregnancy (18 years n = 1833/30 years n = 1593). P-value: Wald’s test for linear tendency, except BMI and WC Wald’s test for heterogeneity. (DOCX) [file pone.0163428.s003.docx]

**S3 Table. Association between FVC and anthropometric variables and body composition (quintiles), males, at 18 and 30 years.**

|  | FVC (L) - Males | | | |
| --- | --- | --- | --- | --- |
|  | 1993 Cohort - 18 years | | 1982 Cohort - 30 years | |
|  | Model 1* | Model 2** | Model 1* | Model 2** |
|  | β (95% CI) | β (95% CI) | β (95% CI) | β (95% CI) |
| BMI | p<0.001 | p<0.001 | p<0.001 | p<0.001 |
| 1^th^ (lowest) | - | - | - | - |
| 2^nd^ | 0.197 (0.117; 0.278) | 0.183 (0.104; 0.263) | 0.171 (0.070; 0.273) | 0.168 (0.063; 0.274) |
| 3^rd^ | 0.229 (0.141; 0.318) | 0.205 (0.116; 0.394) | 0.248 (0.130; 0.366) | 0.253 (0.130; 0.377) |
| 4^th^ | 0.251 (0.144; 0.357) | 0.211 (0.105; 0.318) | 0.185 (0.043; 0.328) | 0.174 (0.025; 0.322) |
| 5^th^ (highest) | 0.144 (-0.025; 0.313) | 0.089 (-0.080; 0.258) | 0.172 (-0.042; 0.385) | 0.162 (-0.058; 0.382) |
| WC | p<0.001 | p<0.001 | p<0.001 | p<0.001 |
| 1^th^ (lowest) | - | - | - | - |
| 2^nd^ | 0.153 (0.075; 0.232) | 0.145 (0.068; 0.223) | 0.116 (0.019; 0.213) | 0.149 (0.048; 0.251) |
| 3^rd^ | 0.194 (0.109; 0.280) | 0.175 (0.089; 0.260) | 0.127 (0.019; 0.235) | 0.141 (0.029; 0.254) |
| 4^th^ | 0.285 (0.187; 0.382) | 0.254 (0.157; 0.351) | 0.040 (-0.085; 0.165) | 0.072 (-0.059; 0.203) |
| 5^th^ (highest) | 0.154 (0.008; 0.300) | 0.128 (-0.018; 0.274) | -0.125 (-0.306; 0.055) | -0.096 (-0.284; 0.091) |
| Triceps skinfold | p<0.001 | p<0.001 | p<0.001 | p= 0.001 |
| 1^th^ (lowest) | - | - | - | - |
| 2^nd^ | -0.001 (-0.077; 0.075) | -0.005 (-0.080; 0.070) | 0.044 (-0.047; 0.135) | 0.036 (-0.059; 0.132) |
| 3^rd^ | -0.043 (-0.121; 0.035) | -0.060 (-0.138; 0.018) | -0.015 (-0.106; 0.076) | -0.032 (-0.127; 0.064) |
| 4^th^ | -0.108 (-0.191; -0.025) | -0.138 (-0.221; -0.055) | -0.087 (-0.180; 0.007) | -0.072 (-0.170; 0.026) |
| 5^th^ (highest) | -0.345 (-0.452; -0.238) | -0.372 (-0.479; -0.265) | -0.139 (-0.239; -0.040) | -0.146 (-0.250; -0.042) |
| Subscapular skinfold | p<0.001 | p<0.001 | p<0.001 | p<0.001 |
| 1^th^ (lowest) | - | - | - | - |
| 2^nd^ | -0.023 (-0.100; 0.054) | -0.048 (-0.125; 0.029) | 0.006 (-0.087; 0.099) | -0.015 (-0.112; 0.083) |
| 3^rd^ | -0.108 (-0.187; -0.029) | -0.110 (-0.189; -0.032) | -0.092 (-0.189; 0.005) | -0.092 (-0.194; 0.010) |
| 4^th^ | -0.090 (-0.176; -0.004) | -0.106 (-0.191; -0.020) | -0.258 (-0.366; -0.151) | -0.250 (-0.363; -0.137) |
| 5^th^ (highest) | -0.323 (-0.438; -0.209) | -0.348 (-0.462; -0.233) | -0.418 (-0.549; -0.287) | -0.417 (-0.554; -0.279) |
| Fat mass BOD POD | p<0.001 | p<0.001 | p<0.001 | p<0.001 |
| 1^th^ (lowest) | - | - | - | - |
| 2^nd^ | -0.079 (-0.153; -0.004) | -0.090 (-0.163; -0.016) | -0.042 (-0.133; 0.047) | -0.057 (-0.151; 0.037) |
| 3^rd^ | -0.190 (-0.267; -0.113) | -0.205 (-0.281; -0.129) | -0.174 (-0.270; -0.078) | -0.165 (-0.267; -0.064) |
| 4^th^ | -0.235 (-0.317; -0.154) | -0.270 (-0.352; -0.189) | -0.345 (-0.448; -0.243) | -0.335 (-0.443; -0.226) |
| 5^th^ (highest) | -0.576 (-0.685; -0.468) | -0.603 (-0.712; -0.494) | -0.683 (-0.815; -0.550) | -0.686 (-0.825; -0.546) |
| Fat-free mass BOD POD | p<0.001 | p<0.001 | p<0.001 | p<0.001 |
| 1^th^ (lowest) | - | - | - | - |
| 2^nd^ | 0.330 (0.243; 0.417) | 0.330 (0.243; 0.417) | 0.339 (0.241; 0.437) | 0.348 (0.245; 0.450) |
| 3^rd^ | 0.378 (0.281; 0.475) | 0.400 (0.302; 0.497) | 0.510 (0.404; 0.617) | 0.518 (0.410; 0.629) |
| 4^th^ | 0.483 (0.379; 0.586) | 0.505 (0.401; 0.609) | 0.639 (0.524; 0.756) | 0.627 (0.504; 0.748) |
| 5^th^ (highest) | 0.563 (0.455; 0.672) | 0.599 (0.491; 0.708) | 0.678 (0.546; 0.811) | 0.675 (0.535; 0.814) |
| Fat mass DXA | p<0.001 | p<0.001 | p<0.001 | p<0.001 |
| 1^th^ (lowest) | - | - | - | - |
| 2^nd^ | -0.032 (-0.110; 0.046) | -0.036 (-0.113; 0.046) | -0.019 (-0.114; 0.075) | -0.019 (-0.118; 0.081) |
| 3^rd^ | -0.062 (-0.143; 0.018) | -0.094 (-0.174; 0.014) | -0.137 (-0.238; -0.035) | -0.143 (-0.251; -0.036) |
| 4^th^ | -0.125 (-0.214; -0.036) | -0.175 (-0.265; -0.085) | -0.269 (-0.384; -0.154) | -0.239 (-0.362; -0.115) |
| 5^th^ (highest) | -0.412 (-0.531; -0.292) | -0.466 (-0.587; -0.345) | -0.464 (-0.599; -0.328) | -0. 470 (-0.616; -0.323) |
| Fat- free mass DXA | p<0.001 | p<0.001 | p<0.001 | p<0.001 |
| 1^th^ (lowest) | - | - | - | - |
| 2^nd^ | 0.274 (0.185; 0.364) | 0.292 (0.203; 0.383) | 0.203 (0.109; 0.298) | 0.249 (0.150; 0.347) |
| 3^rd^ | 0.341 (0.238; 0.445) | 0.372 (0.269; 0.476) | 0.320 (0.216; 0.425) | 0.334 (0.224; 0.443) |
| 4^th^ | 0.389 (0.277; 0.502) | 0.445 (0.331; 0.559) | 0.463 (0.349; 0.578) | 0.481 (0.359; 0.602) |
| 5^th^ (highest) | 0.404 (0.283; 0.525) | 0.474 (0.351; 0.596) | 0.449 (0.314; 0.583) | 0.469 (0.324; 0.613) |
| Trunk fat mass DXA | p<0.001 | p<0.001 | p<0.001 | p<0.001 |
| 1^th^ (lowest) | - | - | - | - |
| 2^nd^ | -0.032 (-0.110; 0.046) | -0.044 (-0.121; 0.033) | -0.009 (-0.103; 0.085) | -0.008 (-0.107; 0.092) |
| 3^rd^ | -0.022 (-0.104; 0.059) | -0.066 (-0.148; 0.016) | -0.141 (-0.243 -0.040) | -0.148 (-0.256; -0.040) |
| 4^th^ | -0.120 (-0.210; -0.031) | -0.164 (-0.255; -0.073) | -0.257 (-0.374; -0.141) | -0.231 (-0.356; -0.106) |
| 5^th^ (highest) | -0.383 (-0.505; -0.261) | -0.454 (-0.578; -0.330) | -0.523 (-0.661; -0.385) | -0.538 (-0.687; -0.389) |
| Arms fat mass DXA | p<0.001 | p<0.001 | p<0.001 | p<0.001 |
| 1^th^ (lowest) | - | - | - | - |
| 2^nd^ | -0.007 (-0.085; 0.071) | -0.026 (-0.103; 0.051) | -0.050 (-0.145; 0.045) | -0.069 (-0.170; 0.032) |
| 3^rd^ | 0.004 (-0.076; 0.083) | 0.039 (-0.119; 0.040) | -0.071 (-0.172; 0.031) | -0.066 (-0.173; 0.041) |
| 4^th^ | -0.123 (-0.212; -0.036) | -0.175 (-0.265; -0.086) | -0.196 (-0.309; -0.083) | -0.203 (-0.325; -0.082) |
| 5^th^ (highest) | -0.363 (-0.478; -0.247) | -0.414 (-0.531; -0.298) | -0.381 (-0.517; -0.245) | -0.392 (-0.537; -0.247) |
| Legs fat mass DXA | p<0.001 | p<0.001 | p<0.001 | p<0.001 |
| 1^th^ (lowest) | - | - | - | - |
| 2^nd^ | -0.025 (-0.103; 0.054) | -0.013 (-0.090; 0.065) | -0.051 (-0.145; 0.043) | -0.040 (-0.139; 0.059) |
| 3^rd^ | -0.045 (-0.126; 0.036) | -0.062 (-0.143; 0.018) | -0.109 (-0.210; -0.009) | -0.099 (-0.206; -0.007) |
| 4^th^ | -0.122 (-0.210; -0.034) | -0.165 (-0.255; -0.076) | -0.203 (-0.311; -0.095) | -0.176 (-0.292; -0.060) |
| 5^th^ (highest) | -0.329 (-0.444; -0.214) | -0.354 (-0.470; -0.239) | -0.348 (-0.471; -0.225) | -0.328 (-0.460; -0.196) |

β: regression coefficient; CI: confidence interval; BMI: body mass index; WC: waist circumference; DXA: dual-energy X-ray absorptiometry; BOD POD: air displacement plethysmography; FVC: forced vital capacity;

DXA variables 18 years: 1778 observations. DXA variables 30 years: 1638 observations.

* Model 1: adjusted by height, weight and skin color (18 years n= 1844 / 30 years n= 1711)

**Model 2: adjusted by model 1 + current asset index (quintiles), current achieved schooling, smoking status, wheezing in the last year, physical activity and corticoids use in the last three months, birth weight and maternal smoking during pregnancy (18 years n=1833/30 years n= 1593).

P-value: Wald’s test for linear tendency, except BMI and WC Wald’s test for heterogeneity.
